# Meeting of the Medical Society of South-Western New York

**Published:** 1857-12

**Authors:** 


*Meeting of the Medical Society of South-Western New York.*—One of the occasions which makes one oblivious to the ills of one's self and the rest of mankind, and which is particularly refreshing at the present time, occurred at Fredonia in the shape of the medical dinner of the Medical Society of South-Western New York; the report comes to us in the Fredonia Censor. The president, Dr. G. W. Hazeltine, welcomed the members and their guests in an appropriate speech, which he concluded with the sentiment,

*The Medical Society of South-Western New York; may it long continue to be what it now is, the pride of those who are worthy to be its members.* This was responded to, in an appropriate manner, by Dr. T. D. Strong, of Westfield. Many other sentiments were proposed, which were well responded to, and among them "The Physician a Hero." We should like to make the acquaintance of Dr. H. M. T. Smith, of Dunkirk, who responded to this toast, for there must be some more left of the genial humor which effervesces in the first part of the Dr.'s speech. He establishes conclusively the connection between real heroism and a hearty dinner, (may he never want one) and brought forward the apt example of "the war between the beef eaters and the rice eaters." "John Bull," he says, "glories in good, sound, hearty roast beef. On a full stomach of his favorite diet, he will put to flight thousands of your poor, hatchet-faced, white-livered barbarians. Gentlemen, I predict that beef will win." The doctors had a good time, and left in good time, banishing, undoubtedly, all visions of troublesome patients and no pay, long midnight rides, etc., etc. F.

